# Transition and identification of pathological states in p53 dynamics for therapeutic intervention

**DOI:** 10.1038/s41598-021-82054-1

**Published:** 2021-01-27

**Authors:** Amit Jangid, Md. Zubbair Malik, Ram Ramaswamy, R. K. Brojen Singh

**Affiliations:** 1 School of Computational and Integrative Sciences, Jawaharlal Nehru University, New Delhi, 110067 India; 2 Department of Chemistry, Indian Institute of Technology Delhi, New Delhi, 110016 India

**Keywords:** Cell biology, Computational biology and bioinformatics, Systems biology

## Abstract

We study a minimal model of the stress-driven p53 regulatory network that includes competition between active and mutant forms of the tumor-suppressor gene p53. Depending on the nature and level of the external stress signal, four distinct dynamical states of p53 are observed. These states can be distinguished by different dynamical properties which associate to *active*, *apoptotic*, *pre-malignant* and *cancer* states. Transitions between any two states, *active*, *apoptotic*, and *cancer*, are found to be unidirectional and irreversible if the stress signal is either oscillatory or constant. When the signal decays exponentially, the apoptotic state vanishes, and for low stress the pre-malignant state is bounded by two critical points, allowing the system to transition *reversibly* from the active to the pre-malignant state. For significantly large stress, the range of the pre-malignant state expands, and the system moves to irreversible cancerous state, which is a stable attractor. This suggests that identification of the pre-malignant state may be important both for therapeutic intervention as well as for drug delivery.

## Introduction

The tumour suppressor gene p53, also termed as the guardian of the genome, crucially determines cell fate through various mechanisms^1,2^. p53 induced different biological outcomes has been studied in detail^4,5^. Activation of the p53 regulatory pathway by internal and external stress can lead to many different outcomes^3,4^. p53 is known to be mutated in cancer, either in an exonic or intronic portion of the gene due to stress^3,6–10^. These mutations eventually lead to disruption in binding DNA. Hence the cell, during transformation, harbours mutated p53 that may finally develop malignancies. From a theoretical network perspective, p53 is a key hub controlling important genes as well as essential cellular functions^1,11,12^. Studies on signalling networks have provided identification of many target genes for therapeutic interventions in the context of cancer^1,11,12^. However, the identification of such genes from a dynamical perspective is still open.

Cancer is a complex disease manifested due to the interaction of non-linear, non-additive, and dissipative components^13^. In order to understand the functionality of the cell in this state, it is required to know its behavior in the normal state and in a perturbed state. Cancer dynamics has been called an emergent property that arises from these interacting components, the constituting genes, small molecules, and the fluctuating environment^14^. p53 holds a central point in signal transduction pathways involving a large number of genes that respond to diverse stress signals^15–18^. This reduces the risk of mutation and prevents circumstances that can lead to cancer or other pathological states^19^. Since the expression and regulation of p53 depend on its interacting partners in the regulatory pathway, it’s modeling often involves both negative and positive feedback mechanisms. Mathematical modeling of the p53 regulatory network can provide dynamical information and patterns to predict cellular mechanisms and its behavior^20, 21^. Still, a challenge is to capture various cellular phases within a simplified minimal model.

Under the influence of MDM2, p53 is maintained at a low level in the normal condition^22^. The emergence of oscillatory behaviour, one such state in p53 dynamics (“active”), has been extensively studied theoretically and experimentally^27–35^. DNA recovery from low dose of ionizing radiation (IR, external stress) corresponds to reversible sustained p53 oscillations with varied amplitude, whereas high dose of IR induces irreversible phase leading to stable state (damped oscillations) which corresponds to apoptosis^16–18,27,28^. The variability in the amplitude of oscillations is found to be larger than the changes in the period of oscillations both for damped and undamped conditions^29^. Further, persistent DNA damage activates ATM, and ATM activates Chk2, which results in p53 oscillations to repair damaged DNA^30^. However, what could be the dynamics of p53 in cancer phase is still a debatable question.

Some models capture various possible dynamical states of p53 which associate to different cell state. It is well known that p53 is coupled with Mdm2 via a negative feedback loop^27–35^. In these studies it was observed that if negative feedback loop gets activated then DNA repair takes place whereas, if positive feedback loop gets activated then p53 activation moves to irreversible apoptotic phase. The other regulators of p53 sometimes regulate p53 pulses, for example, the inclusion of MDMX in the model system suppresses p53 oscillatory amplitude whereas, knocking out MDMX significantly enhances this amplitude^32^. In the recovery phase of damaged DNA, there are repetitive pulses of p53 which are the results of successive efforts of repairing damaged DNA^33^. However, in the case of apoptosis with excess stress, this amplitude of pulse abruptly rises and moves to an irreversible stable state. Similarly, the other regulators of p53 coupled with positive feedback loop (ATM, PTEN, Akt etc) sometimes can induce switching behavior in the p53 dynamical states. Although these models were able to capture these various dynamical states of p53 such as active, recovery and apoptosis which mimic experimental results in a qualitative sense but, could not capture dynamical behavior of p53 in cancer phase.

Once a normal cell becomes cancerous by the mutational process, this signal propagates to neighboring cells^23^, thereby a competition is established between normal and cancer cells^24^. The onset, development and propagation of cancer cell population in the normal cell ecology provides a new transformed physico-chemical state, which bears several similarities to first-order phase transition^25^. A simple “competition” model for cancer is based on two types of cells, normal and cancer with population $N_1$ and $N_2$. Their dynamics in the cellular ecology can be modeled as the following system of equations^26^.

1 $$\begin{aligned} \frac{dN_1}{dt}= & {} R_1N_1\left[ 1-\frac{N_1}{K_1}-C_{12}\frac{N_2}{K_1}\right] \end{aligned}$$

2 $$\begin{aligned} \frac{dN_2}{dt}= & {} R_2N_2\left[ 1-\frac{N_2}{K_2}-C_{21}\frac{N_1}{K_2}\right] \end{aligned}$$

$R_1$, $R_2$ and $K_1$, $K_2$ are the intrinsic growth rates and carrying capacities of species 1 and 2, and $C_{12}$ $(C_{21})$ is the measure of the effect of competition coefficient on species 1(2) by species 2(1). The equilibrium point (critical point $\dot{N_i}$ = 0) of the system is $N_1 = \frac{K_1-K_2 C_{12}}{1-C_{12} C_{21}}$, and $N_2 = \frac{K_2-K_1 C_{21}}{1-C_{12} C_{21}}$. The normal phase corresponds to the condition $\dot{N_1} > 0$, and $\dot{N_2} < 0$ which implies that $C_{12} \le 0$ and $C_{21} \ge {K_2}/{K_1}$ assuming that $N_2<< N_1 = K_1$. Biologically this assumption reflects that in the normal phase, population of cancerous cell is small ($\approx$ 0), while population of normal cells reaches its carrying capacity. The condition $\dot{N_1} < 0$, and $\dot{N_2} > 0$ which implies $C_{12} \ge 0$ and $C_{21} \le {K_2}/{K_1}$ corresponds to cancer progression. Apart from normal and cancer phases, there are important dynamical states which may drive the cellular system into various pathologies. Since this dynamical system is function of various species, $\dot{N_i}=F_i(N_1,N_2,\ldots ,N_m);$ where $i=1 \ldots m$, the exact identification of critical point ($\dot{N_i}$ = 0) is difficult.

In this work, we present a minimal p53 regulatory pathway model to study phase transition like behavior of normal and cancer in the cellular system at molecular level. We also investigate different cancer progression steps captured in the p53 dynamics and the possibility of therapeutic intervention in cancer dynamics. We also discussed various key results in the normal to cancer transition in dynamical sense and observations of various stages of cancer phase.

**Figure 1:**
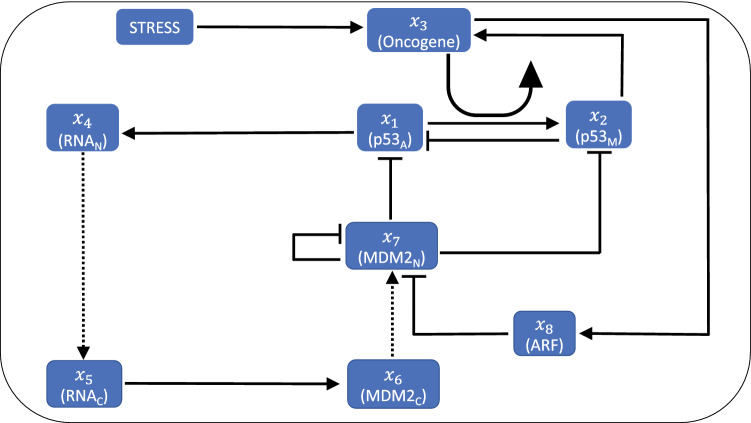
Interaction network for ${\rm p}53_{A}$-${\rm p}53_{M}$-MDM2-ARF-Stress. Dashed arrow shows movement from nucleus to cytoplasm or vice versa, while solid arrow, and bars corresponds to activation, and inhibition on respective node. (Modified network from^35^).

**Table 1:** Parameters values, and descriptions.

S. no.	Parameter	Value	Description	References
1.	$k_{P}$	0.5 proteins/s	[${\rm p}53_{A}$] production	^35^
2.	$k_{1}$	9.963 $\times 10^{-6}$	[MDM2$_{N}$] dependent [${\rm p}53_{A}$] decay	^35^
3.	$d_{P}$	1.925 $\times 10^{-5}$	[${\rm p}53_{A}$] decay	^35^
4.	$\gamma _{_{x_{1}}}$	9.963 $\times 10^{-7}$	[${\rm p}53_{M}$] dependent [${\rm p}53_{A}$] decay	Estimated
5.	$\delta _{_{x_{1}}}$	5.963 $\times 10^{-6}$	[GENE] dependent [${\rm p}53_{A}$] decay	Estimated
6.	$K_{1}$	50	Cooperative coefficient	Estimated
7.	$n_{1}$	4	Hill coefficient	Estimated
8.	$\alpha _{_{x_{2}}}$	1.5 $\times 10^{-7}$ proteins/s	[${\rm p}53_{M}$] production	Estimated
9.	$\gamma _{_{x_{2}}}$	9.963 $\times 10^{-6}$	[MDM2$_{N}$] dependent [${\rm p}53_{M}$] decay	Estimated
10.	$\delta _{_{x_{2}}}$	1.925 $\times 10^{-5}$	[${\rm p}53_{M}$] decay	Estimated
11.	$\alpha _{_{x_{3}}}$	1.5 $\times 10^{-7}$	[GENE] production	Estimated
12.	$\beta _{_{x_{3}}}$	6.5 $\times 10^{-3}$	Stress dependent maximum [GENE] activation rate	Estimated
13.	$K_{2}$	3	Cooperative coefficient	Estimated
14.	$n_{2}$	3	Hill coefficient	Estimated
15.	$\delta _{_{x_{3}}}$	2.4375 $\times 10^{-3}$	${\rm p}53_{M}$ dependent maximum [GENE] activation rate	Estimated
16.	$K_{3}$	1000	Cooperative coefficient for oncogene	Estimated
17.	$n_{3}$	3	Hill coefficient	Estimated
18.	$\gamma _{_{x_{3}}}$	1.925 $\times 10^{-5}$	[GENE] decay	Estimated
19.	$k_{m}$	1.5 $\times 10^{-3}$	[RN${\rm A}_{N}$] production	^35^
20.	$k_{2}$	1.5 $\times 10^{-2}$	[${\rm p}53_{A}$] dependent maximum [RN${\rm A}_{N}$] activation rate	^35^
21.	$k_{D}$	740.0	Cooperative coefficient	^35^
22.	$k_{0}$	8.0 $\times 10^{-4}$	[RN${\rm A}_{N}$] decay and [RN${\rm A}_{C}$] production	^35^
23.	$d_{rc}$	1.444 $\times 10^{-4}$	[RN${\rm A}_{C}$] decay	^35^
24.	$k_{T}$	1.66 $\times 10^{-2}$	[MDM2$_{C}$] production	^35^
25.	$k_{i}$	9.0 $\times 10^{-4}$	[MDM2$_{C}$] decay and [MDM2$_{N}$] production	^35^
26.	$d_{mn}$	1.66 $\times 10^{-7}$	[MDM2$_{N}$] decay	^35^
27.	$k_{3}$	9.963 $\times 10^{-6}$	[ARF] dependent [MDM2$_{N}$] decay	^35^
28.	$k_{a}$	0.5 proteins/s	[ARF] production	^35^
29.	$K_{4}$	10	Cooperative coefficient for ARF	Estimated
30.	$n_{4}$	3	Hill coefficient	Estimated
31.	$\delta$	3.5 $\times 10^{-6}$	[ARF] activation rate due to stress	Estimated
32.	$d_{a}$	3.209 $\times 10^{-5}$	[ARF] decay	^35^
33.	$k_{3}$	9.963 $\times 10^{-6}$	[MDM2$_{N}$] dependent [ARF] decay	^35^

## Materials and methods

### Minimal p53_A_-p53_M_**MDM2-ARF regulatory network model**

The proposed model is a minimal regulatory network $\hbox {p53}_{\rm A}-\hbox {p53}_{\rm M}$-MDM2-ARF under stress condition. This involves the interaction of activated p53 ($\hbox {p53}_{\rm A}$) and mutated p53 ($\hbox {p53}_{\rm M}$) along with other key regulators MDM2, ARF and related molecular species as shown in Fig. 1. In the model^35^, p53 induces transcription of $\hbox {RNA}_{\rm N}$ and is also produced in the nucleus with a constant basal rate. After being produced, it is translated at a constant rate after proceeding in the cytoplasm, and this, cytoplasmic RNA, is followed by eventual decay. Cytoplasmic MDM2 is transported to the nucleus, where it regulates p53 via negative feedback in three different ways. First, transcriptional activity by binding to the p53 transactivation domain^36^, second it promotes p53 degradation^37,38^, and finally it favours the export of p53 from the nucleus to the cytoplasm^39^.

Oncogene activation can be incorporated in the model through either structural alterations (such as chromosomal rearrangement, mutation) or epigenetic modification (gene promoter hypomethylation)^40^. In the present model, we have assumed that a certain type of stress signal *S*, which is capable of causing the structural, and epigenetic modification that results in the activation of oncogenes. The activated oncogene then activates ARF within the nucleus^41,42^ and since ARF is a direct inhibitor of MDM2 activity by binding to the RING finger domain of MDM2 this sequesters MDM2^43^. Tao, and Levine has observed that ARF blocks the nucleo-cytoplasmic shuttling of MDM2, which is essential for the ability of MDM2 to export p53 into the cytoplasm^44^.

Weber and others showed that ARF binds to MDM2 gene and sequesters it into the nucleolus, which in turn prevents p53 regulation by MDM2, and hence leads to the activation of p53^45^. ARF gets activated due to activation of different oncoproteins such as Myc, Ras, and EIA^45–48^. It is now well known that activated oncogene, such as c-Myc, leads to the promotion of mutant p53^49^, and this mutated p53 induces the expression of oncogenes^50,51^ as well as inhibits the activity of activated p53 to prevent the cell apoptosis^52–56^. ARF moves from nucleus to cytoplasm to bind the MDM2 and releases the p53 which is due to activation of oncogene^57^.

We incorporate the regulating activity of an oncogene in the p53 network model. In a recent study, it has been shown that regulation/deregulation of c-Myc expression due to stress signal can induce mutation/s in the expression of p53 by binding to CA(C/T)GTG-containing site in the p53 promoter^58^. Hence, it has been suggested that stress-induced deregulation of c-Myc expression could increase the expression of mutated p53. On the other hand, it has been observed that mutant p53 can regulate c-Myc expression by activating c-Myc promoter through C-terminus^50^. Further, it has been reported that p53 repress the c-Myc expression by inducing tumor suppressor miR-145^59^, because c-Myc repression by p53 is required to control the G1 cell cycle arrest^60^, such that activation of c-MYC allows the functioning of mutant p53^61^. Hence, the oncogene we have incorporated in the model is of c-Myc (Myc) type which allows interacting $\hbox {p53}_{\rm M}$ with an oncogene, and we studied the dynamical behavior of the model system which gives the similar behavior as the main model (Fig. S1). To keep the model simple, we have used either hill function or direct interaction between different proteins, and parameters are estimated to observe the qualitative behavior. Parameters values, and descriptions are given in Table 1.

### Mathematical framework of the model system

In the proposed model system (Fig. 1) can be represented by a state vector, $\vec {X}=[x_1,x_2,\ldots ,x_8]^\intercal$, where, $\intercal$ is the transpose of the vector, and $x_i; i=1,2,\ldots ,8$ represents the concentrations of the corresponding molecular species such that $\vec {X}$=$[p53_A, p53_M, Oncogene, RNA_{N}, RNA_{C}, MDM2_{C}, MDM2_{N}, ARF]^\intercal$. Then the model regulatory network is perturbed with stress with strength *S*, which could be irradiation (IR), molecular (or chemical toxic) fluctuations, environmental fluctuations etc. The amount of stress imparted in the model depends on the *S* strength, and nature of the *S* form introduced in the system. In this work, we have taken three different types of nature of stress *S*, 1) constant stress form $S = I$, 2) periodic stress $S = I (1 + sin(2\pi t))/T$, ($T=6$ h throughout the model ), and 3) exponentially decaying stress $S = I e^{-\lambda t}$. Constant and sinusoidal stress can be considered as a type of chronic stress allowing continuous exposure of stress-causing unabated production of stress hormones which could be the cause of cancer^62^. Some types of stress-causing sinusoidal signals are low energy radiofrequency signals may cause DNA damage which might lead to cancer phase^63,64^. Decaying stress could of the form of radioactive radiation which might cause cancer^65^. Based on the proposed model system, we arrived at the following set of coupled ordinary differential equations,

$$\begin{aligned} \frac{dx_1}{dt}= & {} k_{p} - \left( k_{1} x_7 + d_{p} + \gamma _{_{x_1}} x_2 + \delta _{_{x_1}} \frac{x_3^{n_{1}}}{K_{1}^{n_{1}}+x_3^{n_{1}}} \right) x_1\\ \frac{dx_2}{dt}= & {} \alpha _{x_2} + \delta _{x_1} \frac{x_3^{n_{1}}}{K_{1}^{n_{1}}+x_3^{n_{1}}} x_1 - \gamma _{x_2} x_7 x_2 - \delta _{x_2} x_2\\ \frac{dx_3}{dt}= & {} \alpha _{x_3} + \beta _{x_3} \frac{S^{n_{2}}}{K_{2}^{n_{2}} + S^{n_{2}}} + \delta _{x_3} \frac{{x_2}^{n_{3}}}{K_{3}^{n_{3}} + {x_2}^{n_{3}}} - \gamma _{x_3} x_3\\ \frac{dx_4}{dt}= & {} k_{m} + k_{2} \frac{x_1^{1.8}}{k_{D}^{1.8}+x_1^{1.8}} - k_{0} x_4\\ \frac{dx_5}{dt}= & {} k_{0} x_4 - d_{rc} x_5\\ \frac{dx_6}{dt}= & {} k_{T} x_5 - k_{i} x_6\\ \frac{dx_7}{dt}= & {} k_{i} x_6 - d_{mn} x_6^2 - k_{3} x_7 x_8\\ \frac{dx_8}{dt}= & {} k_{a} + \delta \frac{x_3^{n_{4}}}{K_{4}^{n_{4}} + x_3^{n_{4}}} x_8 - d_{a} x_8 - k_{3} x_7 x_8 \end{aligned}$$

Here, $k_{p}$ represents the production rate of $\hbox {p53}_{\rm A}$, $k_{1}$ is the rate at which $\hbox {MDM2}_{\rm N}$ ubiquitinates $\hbox {p53}_{\rm A}$, $d_{p}$ is the degradation rate of $\hbox {MDM2}_{\rm N}$ independent of $\hbox {p53}_{\rm A}$, $\gamma _{x_{1}}$ is the degradation rate due to $\hbox {p53}_{\rm M}$ inhibition. Further, $\delta _{x_{1}}$ shows the rate of mutation in $\hbox {p53}_{\rm A}$ into $\hbox {p53}_{\rm M}$ due to pro-oncogene (oncogenic mutation), $n_{1}$ is Hill coefficient, and $K_{1}$ is the dissociation constant. $\alpha _{x_2}$ is the production rate of $\hbox {p53}_{\rm M}$ independent from pro-oncogene (which can be ignored), $\delta _{x_{1}}$ is mutational translation rate of $\hbox {p53}_{\rm A}$ into $\hbox {p53}_{\rm M}$ due to pro-oncogene mutation. Now, $\gamma _{x_2}$ is inhibition due to $\hbox {MDM2}_{\rm N}$ ($\hbox {MDM2}_{\rm N}$ dependent degradation), and $\delta _{x_2}$ shows natural degradation rate of $\hbox {p53}_{\rm M}$ (which can be ignored). $\alpha _{x_{3}}$ is the production rate of pro-oncogene (ONCO) independent of stress (which can be ignored), $\beta _{x_{3}}$ is the stress dependent activation rate of pro-oncogene (oncogene), $n_{2}$ hill coefficient, $K_{2}$ is dissociation constant, $\delta _{x_{3}}$ is the mutated $\hbox {p53}_{\rm M}$ dependent activation rate, $n_{3}$ is Hill coefficient, $K_{3}$ is the dissociation constant, and $\gamma _{x_{3}}$ is the natural degradation rate. $k_{m}$ represents the production rate of nucleic mRNA, $k_{2}$ is the maximum production rate of nucleic mRNA, $K_{D}$ represents dissociation parameter for p53, and $k_{0}$ is the transportation rate of nucleic mRNA into cytoplasm. $d_{rc}$ represents decay rate of mRNA into cytoplasm. $k_{T}$ represents the translation rate of $\hbox {MDM2}_{\rm C}$, while $k_{i}$ represents nuclear localization of $\hbox {MDM2}_{\rm C}$. $d_{mn}$ is the rate of MDM2 auto ubiquitination, and $k_{3}$ is the degradation rate of $\hbox {MDM2}_{\rm N}$ due to binding ARF to $\hbox {MDM2}_{\rm N}$. $k_{a}$ is the production rate of ARF, $\delta$ is the maximum activation rate of ARF due to pro-oncogene activation, $n_{4}$ is hill coefficient, $K_{4}$ is the dissociation constant, $d_{a}$ is the natural degradation rate of ARF, and $k_{3}$ is the $\hbox {MDM2}_{\rm N}$ dependent degradation of ARF.

## Results

The system of coupled ordinary differential equations is numerically integrated using ODEINT Python. Numerical simulations are carried out for an arbitrary set of initial values for the variables, and after discarding transients, the system dynamics is examined. Initial values of mutant p53 and oncogene are kept zero for each form of the new stress discussed earlier assuming there are no mutant p53, and oncogene initially. As the system is eight-dimensional, so it is hard to explore the whole space of initial conditions. Still, we have taken a large range of initial conditions where we are predicting similar behavior. So we believe that the system is robust for different initial conditions.

### Phase transition driven by Stress

**Figure 2:**
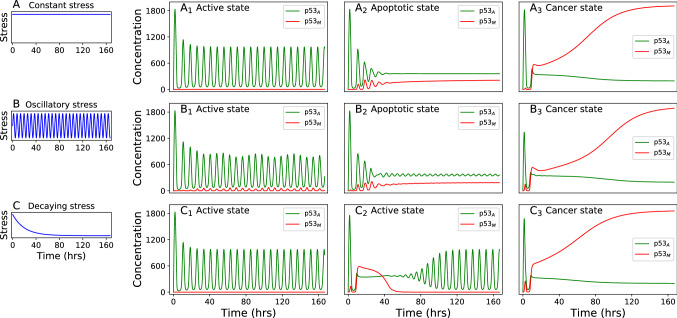
The left column show three different form of stress discussed about. In the top row (**A**_**1**_) (normal), (**A**_**2**_) (apoptosis), and (**A**_**3**_) (cancer) display the time course of $\hbox {p53}_{\rm A}$ (green), and $\hbox {p53}_{\rm M}$ (red) for constant stress of magnitude 1.00, 1.75, and 2.50 respectively ($K_3$=1000.0). (**B**_**1**_) (normal), (**B**_**2**_) (apoptosis), and (**B**_**3**_) (cancer) display the time series for averaged oscillatory stress of magnitude 1.00, 1.50, and 2.50 respectively ($K_3$=1000.0). And (**C**_**1**_) (normal), (**C**_**2**_) (recovery from initial cancer stage), and (**C**_**3**_) (cancer) display the time series for decaying stress of magnitude 1.00, 3.5, and 4.50 respectively ($K_3$=500.0 and $\lambda$=0.05 h$^{-1}$).

Figure 2$A_1$, $A_2$, and $A_3$ show the time course of $\hbox {p53}_{\rm A}$ and $\hbox {p53}_{\rm M}$ for constant stress signal for three different magnitudes $I=1.0$, $I=1.75$, and $I=2.5$ respectively. For small magnitude of stress signal ($S=1$, Fig. 2${\rm A}_1$) both $\hbox {p53}_{\rm A}$ and $\hbox {p53}_{\rm M}$ dynamics show sustained oscillations in which amplitude of oscillations of activated p53 is very high in contrast, the amplitude of mutated p53 is negligibly small. This scenario indicates the possibility of repairing damaged DNA induced by stress signal via p53-MDM2. In such situation, repetitive pulses of $\hbox {p53}_{\rm A}$, which dominate those of $\hbox {p53}_{\rm M}$ in the system, will be generated if damaged DNA is not properly repaired after delivering the first pulse. Once the stress is removed, the cell comes to the normal state. Hence sustained oscillations of $\hbox {p53}_{\rm A}$ may correspond to the repeated repair efforts of the system to fix damaged DNA.

If the magnitude of the stress signal is significantly high ($I=1.75$ Fig. 2${\rm A}_2$ ). The system attempts to repair damaged DNA by generating few pulses (five) of activated p53 (indicated by damped oscillations in $\hbox {p53}_{\rm A}$ and $\hbox {p53}_{\rm M}$ dynamics). This could be the indication that after first pulse, the system sees that the damage is not repairable, it delivers the followed pulses with smaller amplitudes, and moves to amplitude death state^66^, $\hbox {A}_{{\rm p53}_{A}}$ $\rightarrow$ 0, $\hbox {A}_{{\rm p53}_{M}}$ $\rightarrow 0$ (when the cell dies out due to apoptosis) with $\hbox {p53}_{\rm A}$ > $\hbox {p53}_{\rm M}$. Then p53 pathway activates many apoptogenic genes, by delivering a constant pulse of activated p53 (accumulation of active p53), to kill the cell before mutated p53 gets control over the $\hbox {p53}_{\rm A}$ at stress condition^67,68^. Alternatively, p53 can also trigger apoptosis by inhibiting antiapoptotic genes (surviving), thus promoting caspase activation^69^. This phase corresponds to apoptotic phase (amplitude death^66^ after damped oscillations), where the concentration of $\hbox {p53}_{\rm A}$ still dominates that of $\hbox {p53}_{\rm M}$ in the cellular dynamics.

In the third phase $\hbox {p53}_{\rm A}$ and $\hbox {p53}_{\rm M}$ dynamics, for hight stress ($S=2.5$), are different from earlier two phases (Fig. 2$\hbox {A}_{3}$). In this phase, $\hbox {p53}_{\rm M}$ concentration grows rapidly, and is high compared to $\hbox {p53}_{\rm A}$ in the normal phase, indicating uncontrolled behavior of $\hbox {p53}_{\rm M}$. This dynamical behavior is qualitatively similar to the experimental observation of higher expression of mutated p53 in cancer cell and in some conditions, mutated p53 has dominated effect over active p53^70,71^. The *normal to cancer* transition (NCT) is irreversible: the stress *S*, imparted to the system, is able to drive the system into three such distinct dynamical states *active, apoptosis* (indicated by dominant $\hbox {p53}_{\rm A}$, and low $\hbox {p53}_{\rm M}$) and *cancer* ($\hbox {p53}_{\rm M}$ concentration rapidly increasing behavior, and low concentration of $\hbox {p53}_{\rm A}$ with slow decay) states (Fig. 2).

We studied the system dynamics driven by periodic stress of magnitude $I=1.0, 1.5, 2.5$ (Fig. 2$\hbox {B}_{1}$, $\hbox {B}_{2}$ and $\hbox {B}_{3}$ respectively). We observed three distinct dynamical phases, active, apoptosis, and cancer phase (Fig. 2$\hbox {B}_{1}$, $\hbox {B}_{2}$ and $\hbox {B}_{3}$ respectively), which are qualitatively similar to the constant stress case (Fig. 2$\hbox {A}_{1}$, $\hbox {A}_{2}$ and $\hbox {A}_{3}$ respectively). However, the behavior of $\hbox {p53}_{\rm A}$ and $\hbox {p53}_{\rm M}$ in Fig. 2$\hbox {B}_{2}$, after successive four pulses (with decaying pulses amplitudes), we still observed oscillations with a small amplitude which do not die out with time which is negligible to the oscillations in compare of an active state. Increasing the magnitude, this oscillatory behavior dies out (not shown here). In the case of cancer phase, the monotonical growth of $\hbox {p53}_{\rm M}$ is a little slower as compared to constant stress signal case indicating periodic signal helps the cell to prevent moving to either apoptosis or cancer phase.

**Figure 3:**
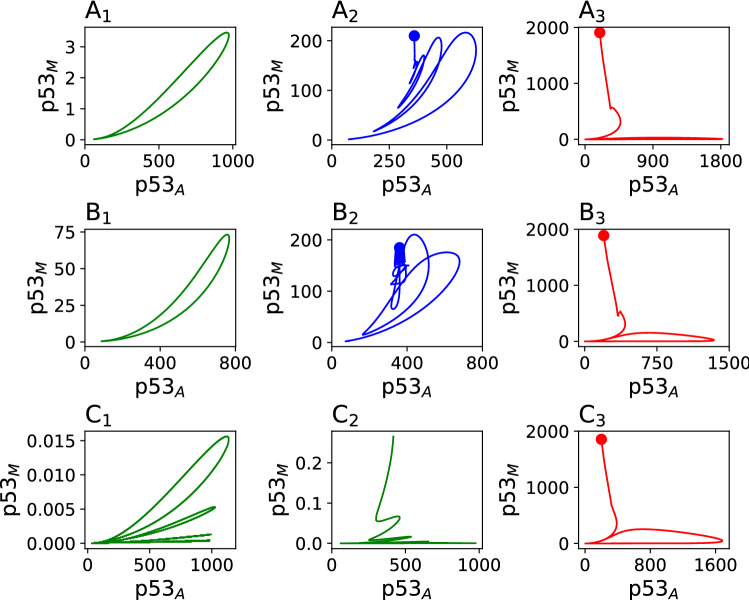
Dynamics on the phase plane for the time series results in the Fig. 2. Green color indicates active state, blue color apoptotic, and red color cancer state. The dot shows the attractor (end point of the trajectory).

The scenario of the behavior of the system dynamics is different in the case of exponentially decay stress. Figure 2$\hbox {C}_{1}$, $\hbox {C}_{2}$ and $\hbox {C}_{3}$ show the time course of $\hbox {p53}_{\rm A}$, and $\hbox {p53}_{\rm M}$ for the magnitude $I=1.0, 3.5,4.5$. For $I=1.0$, we observed an active state with sustain oscillations (Fig. 2$\hbox {C}_{1}$). Increasing the stress ($I=3.5$), the dynamics shows that first the stress provides a shock to the system allowing $\hbox {p53}_{\rm A}$ move to amplitude death^66^ ($\hbox {A}_{{\rm p53}_{A}}$ $\rightarrow$ constant) for small interval of time $T_{ps} \rightarrow$ [9.8–37] h, whereas $\hbox {p53}_{\rm M}$ concentration is suddenly increased dominating $\hbox {p53}_{\rm A}$ concentration during $T_{ps}$. Since $\hbox {p53}_{\rm M}$ dominates over $\hbox {p53}_{\rm A}$ during $T_{ps}$, this state could be considered as a *premalignant signature* of the system dynamics which can be termed as *critical state*^25^. During this short time interval ($T_{ps} \rightarrow$ finite and $\hbox {A}_{{\rm p53}_{A}}$ $\rightarrow$ constant), the active state of the system is collapsed, and $\hbox {p53}_{\rm M}$ gets dominated, and if $T_{ps}\rightarrow \infty$, then the system moves towards cancer phase. Identification of this *critical state* in cancer patients is very crucial for possible therapeutic intervention for preventing from cancer. After this time interval, the system regains its active state, where, $p53_A$ attains it’s oscillating state by suppressing $\hbox {p53}_{\rm M}$ concentration level, and then the system repairs damaged DNA. Significantly high dose of the stress signal triggers higher expression of mutated p53 protein than activated p53 which corresponds to the cancer phase. Hence, in case of exponentially decaying stress signal, we are able to observe only two phases active, and cancer phase. Dynamics on the phase plane, for the time series used in the Fig. 2, are shown in Fig. 3. Green color indicates active state (${\rm A}_1$, ${\rm B}_1$, ${\rm C}_1$, and ${\rm C}_2$), blue color apoptotic (${\rm A}_2$, and ${\rm B}_2$), and red color cancer state (${\rm A}_3$, ${\rm B}_3$, and ${\rm C}_3$). The dot denotes the attractor (end point of the trajectory).

### Oncogenic regulation of normal and cancer dynamics

**Figure 4:**
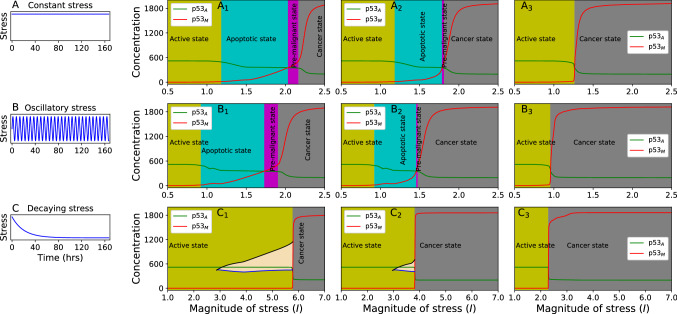
The left column show three different form of stress discussed about. ${\rm A}_{1}$, ${\rm A}_{2}$, and ${\rm A}_{3}$ display the steady state behaviour against magnitude of stress for different K$_{3}$ values 1000.0, 500.0, and 100.0 respectively driven with constant stress. ${\rm B}_{1}$, ${\rm B}_{2}$, and ${\rm B}_{3}$ display the steady state behaviour against magnitude of stress for different K$_{3}$ values 1000.0, 500.0, and 100.0 respectively driven with oscillatory stress. ${\rm C}_{1}$, ${\rm C}_{2}$, and ${\rm C}_{3}$ display the steady state behaviour against amplitude for different K$_{3}$ values 1000.0, 500.0, and 100.0 respectively driven with decaying stress. Yellow region, cyan region, and grey region correspond to active, apoptotic, premalignant, and cancer state respectively. In panel ${\rm C}_{1}$, and ${\rm C}_{2}$ (wheat region) black line (upper line), and blue line (lower line) show maximum of ${\rm p}53_{M}$, and maximum of ${\rm p}53_{A}$ in T$_{ps}$ (see the text) time region, which corresponds to the initial cancer condition.

In this section, we study the cooperative impact of an oncogene on the dynamics of $\hbox {p53}_{\rm A}$ and $\hbox {p53}_{\rm M}$ in the regulating pathway. We consider microscopic dissociation parameter $K_3$, which is an equilibrium constant that amounts to the probability per unit time to dissociate molecular complex^72^. Figure 4 shows steady-state behavior of $\hbox {P53}_{\rm A}$, and $\hbox {p53}_{\rm M}$ as a function of magnitude of stress (*I*) for three different values of $K_3=1000,500,100$. The system’s behavior and transition of the states can be studied from steady-state behavior (Fig. 4). For oscillatory behavior of $\hbox {p53}_{\rm A}$ and $\hbox {p53}_{\rm M}$, the mean population is the average of the maxima and minima of the oscillation calculated in the time window of $t=145.82hr-166.66hr$ (removing transients) whereas, in case of no oscillations, population of $\hbox {p53}_{\rm A}$, and $\hbox {p53}_{\rm M}$ at time 166.66 h were taken as stable fixed point (endpoint of the trajectory).

We observed different phases/states (Fig. 4$\hbox {A}_{1}$, $\hbox {A}_{2}$ and $\hbox {A}_{3}$) in the dynamics of $\hbox {p53}_{\rm A}$ and $\hbox {p53}_{\rm M}$ driven by constant stress for three different values of $K_3=1000,500,100$. For small *I* values ($I \langle 1.19$), the criteria for this was as average value of $\hbox {p53}_{\rm A}$ reduces by 5$\%$ to its maximum averaged value in the case of without stress, both $\hbox {p53}_{\rm A}$ and $\hbox {p53}_{\rm M}$ exhibit oscillatory behavior (Fig. 4$\hbox {A}_{1}$, $K_3=1000$) with the concentration of $\hbox {p53}_{\rm M}$ maintained at minimum level as compared to that of $\hbox {p53}_{\rm A}$. This phase may be considered as active phase (yellow region) of the cellular system, where, $\hbox {p53}_{\rm A}$ delivers successive pulses to activate various genes which are involved in the pathway to repair damaged DNA. In this case, one can see that difference between $x_1$ and $x_2$ (any two points in the trajectory of $\hbox {p53}_{\rm A}$ and $\hbox {p53}_{\rm M}$ in one parameter space respectively) is almost constant ($\Delta x_{12} \rightarrow constant$). Increasing the strength of the stress *I* ($I \rightarrow [1.19{-}2.04]$), we observe that $\Delta x_{12}$ becomes variable where, $\hbox {p53}_{\rm A}$ > $\hbox {p53}_{\rm M}$ and $\hbox {A}_{{\rm p53}_{{\rm A}}}$, $\hbox {A}_{{\rm p53}_{\rm M}}$ $\rightarrow$ *constant* exhibits amplitude death (cell programmed death) scenario in both $\hbox {p53}_{\rm A}$ and $\hbox {p53}_{\rm M}$ dynamics. This state may correspond to apoptotic state (cyan region) in the system dynamics.

In apoptotic phase, the system is not able to repair damaged DNA thereby, $\hbox {p53}_{\rm A}$ activates apoptogenic genes favoring to program cell death. It can also be observed that the concentration levels of both $\hbox {p53}_{\rm A}$ and $\hbox {p53}_{\rm M}$ are converged to a *critical* level $x_c$ as $I \rightarrow I_c=2.04$, which is termed as *critical point* (Fig. 4$\hbox {A}_{1}$). This *critical point* can be defined such as: $\displaystyle \lim _{I\rightarrow I_c}\Delta x_{12}\rightarrow 0$ and $x_1,x_2\rightarrow x_c$. Slight increase in *I* ($I > I_c = 2.04$) triggers slow dominance of $\hbox {p53}_{\rm M}$ over $\hbox {p53}_{\rm A}$, which is the beginning of new departure to the cancer phase. This new stage can be termed as pre-malignant regime (magenta area). Further increasing *I*, $\hbox {p53}_{\rm M}$ is found to rapidly increased, while $\hbox {p53}_{\rm A}$ is decreased significantly low, indicating $\hbox {p53}_{\rm A}$ can no longer control $\hbox {p53}_{\rm M}$ signal such that $\Delta x_{12}$ rapidly increased and then becomes stable. Hence, this phase may be considered as cancer phase (grey area)^25, 73^. In this case, critical point can be seen as the point of departure to either in apoptotic phase or cancer phase.

**Figure 5:**
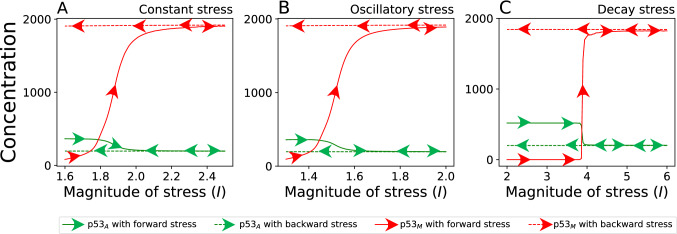
Hysteresis plot for three different type of stress as discussed in main text. (**A**) Increasing the magnitude of stress, apoptotic state moves towards cancer phase while decreasing stress, the cancer phase does not come back to apototic phase. (**B**) and (**C**) show similar pattern for oscillatory and decaying stress respectively. Solid and dashed arrow correspond to increasing and decreasing stress magnitude respectively. For solid arrow the initial value of variable corresponds to apoptotic phase while dashed arrow correspond to cancer phase. Parameters used in the figure correspond to the parameters used in Fig. 4${\rm B}_2$, ${\rm C}_2$, and ${\rm C}_2$ respectively.

Decreasing the value of dissociation parameter $K_3=500$, we observe similar behavior in $\hbox {p53}_{\rm A}$ and $\hbox {p53}_{\rm M}$ dynamics (Fig. 4$\hbox {A}_{2}$), but *critical point* can be obtained at smaller value of magnitude of stress signal, $I_c=1.79$ and range of apoptotic and pre-malignant state get shrinked and the range of cancer phase increased as compared to the case $K_3=1000$ (Fig. 4$\hbox {A}_{1}$). For comparatively small value of dissociation parameter $K_3=100$, $\Delta I_{ms} \approx 0$ and $\Delta I_{cs}\rightarrow large$ (Fig. 4$\hbox {A}_{3}$). In such situation, a stress state suddenly moves from active to cancer phase crossing critical point without showing the signatures of apoptotic and pre-malignant states, and then become steady ($\Delta x_{12}\rightarrow constant$) both in $\hbox {p53}_{\rm A}$ and $\hbox {p53}_{\rm M}$. It may lead to first order phase transition. In case of c-Myc we did not observe pre-malignant regime In constant stress case (supplementary information, Fig. 2$\hbox {A}_{1}$, $\hbox {A}_{2}$, and $\hbox {A}_{3}$).

In case of periodic stress, and for same values of $K_3=1000,500,100$ (Fig. 4$\hbox {B}_{1}$, $\hbox {B}_{2}$ and $\hbox {B}_{3}$ respectively), we observed the similar pattern of four states along with critical point as we found in the case of constant stress. This results also show that all the four states can be obtained at significantly smaller values of stress signal *I* as compared to those of constant stress case. We observed different scenario for exponentially decay stress. In this case, we only observe three states, active, pre-malignant and cancer state for $K_3=1000,500$ and only two states (Fig. 4$\hbox {C}_{1}$, $\hbox {C}_{2}$ and $\hbox {C}_{3}$ respectively) for $K_3=100$. We have also observed that there are two critical points, $x_{c_1}$ and $x_{c_2}$ ($x_{c_2} > x_{c_1}$) in the range $\Delta I=I_{c_2}-I_{c_1}$ (wheat region, Fig. 4 panels $\hbox {C}_{1}$ and $\hbox {C}_{2}$). In this range $\Delta I$, $\hbox {p53}_{\rm M}$ dominates over $\hbox {p53}_{\rm A}$ for a certain time interval $T_{ps}$ (previous section), which is a signature of pre-malignant or critical state, which comes back to the active state after $T_{ps}$ time interval if $I \in [I_{c_1},I_{c_2}]$, where, $I_{c_2}=5.80$ for $K_3=1000$ and $I_{c_2}=3.83$ for $K_3=500$. In the dynamical system study, the identification of this *critical point/s* and *pre-malignant* regime of any cancer type are quite important for therapeutic intervention of the cancer^25^. The reason could be if system dynamics is in this regime $I\in [I_{c_1},I_{c_2}]$, there is a possibility of repairing damaged DNA. For lower value of $K_3$ parameter ($K_3=100$), if $I > I_{c_2}$ the two critical points become single $I_{c_1}=I_{c_2} > I_c$, and the active state directly jumps to cancer state ($T_{ps}\rightarrow \infty$) via $I_c$ (Fig. 4$\hbox {C}_{3}$). These critical points can be seen as the points of departure to either in active state or cancer state. All these results indicate that the impact of oncogene is quite significant in regulating normal and cancer dynamics as well as their state transition. Similar behavior was observed in case of c-Myc as oncogene for constant stress Fig. S2${\rm A}_1$,${\rm A}_2$,${\rm A}_3$ (for three different K$_3$ values), for oscillatory stress Fig. S2${\rm B}_1$,${\rm B}_2$,${\rm B}_3$ (for three different K$_3$ values), and for decaying stress Fig. S2${\rm C}_1$,${\rm C}_2$,${\rm C}_3$ (for three different K$_3$ values).

In the purposed model, we have observed three main phases, recovery, apoptotic and cancer. From dynamical perspective, recovery phase corresponds to a stable limit cycle (Fig. 3${\rm A}_1$,${\rm B}_1$,${\rm C}_1$), apoptotic, and cancer phase correspond to a stable fixed point ($\lambda _{largest}$ = -0.0066, $-0.0072, -0.0714, -0.0714, -0.0695$ for Fig. 1${\rm A}_2$,${\rm B}_2$,${\rm A}_3$,${\rm B}_3$,${\rm C}_3$ respectively )^74^. We observed that increasing the magnitude of stress leads a cell state into a different state. Starting from apoptotic phase, $\hbox {p53}_{\rm A}$ > $\hbox {p53}_{\rm M}$ and $\hbox {p53}_{\rm A}$, $\hbox {p53}_{\rm M}$ $\rightarrow$ *constant*, (taking initial condition of variables such that the dynamics of the system lead to apoptotic phase), increasing magnitude of stress moves apoptotic phase towards cancer phase ($\hbox {p53}_{\rm M}$ > $\hbox {p53}_{\rm A}$ and $\hbox {p53}_{\rm A}$, $\hbox {p53}_{\rm M}$ $\rightarrow$ *constant*) (Fig. 5 solid arrow). A similar scenario was observed for oscillatory and decaying stress (Fig. 5B,C solid arrow). As we start from cancer phase, taking initial condition of variables as it leads to cancer phase, decreasing the magnitude of stress $\hbox {p53}_{\rm A}$, and $\hbox {p53}_{\rm M}$ do not follow the same path (Fig. 5 dashed arrow), which shows irreversibility of apoptotic phase from cancer phase. A similar scenario has been observed in the case of oscillatory and decaying phase. Figure 5B,C shows irreversibility from cancer phase to apoptotic and recovery phase, respectively, as observed in the real picture of cancer. Similar scenario was observed transition between recovery and apoptotic state where, stress in forwarding direction moves recovery state into apoptotic phase whereas, backward stress does not move apoptotic phase into recovery phase, which shows irreversible transition from apoptotic phase to the recovery phase.

#### Phase transition and key to therapeutic intervention

**Figure 6:**
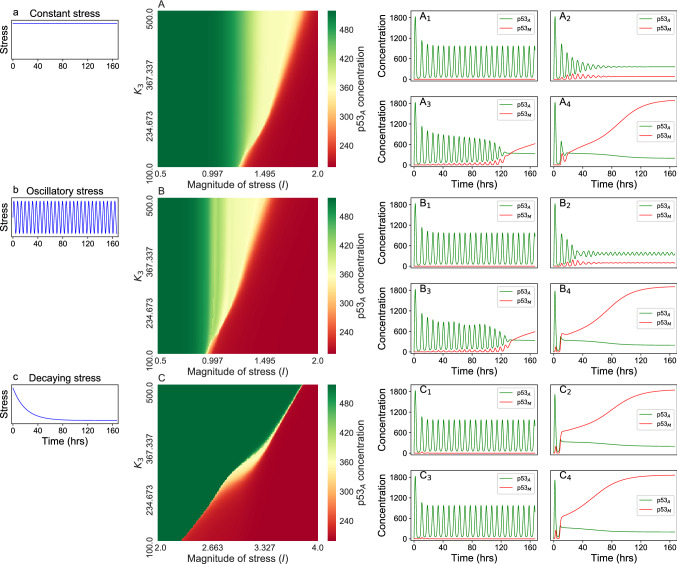
The left column show three different form of stress discussed about. Second column (**A**–**C**) show two parameter steady state behavior of the system for three different form of driven stress (**a**–**c**) respectively. ${\rm A}_1$, ${\rm A}_2$, ${\rm A}_3$, and ${\rm A}_4$ correspond to the time course of $\hbox {p53}_{\rm A}$ (green), and $\hbox {p53}_{\rm M}$ (red) for the parameter set (0.5,500.0), (1.6, 500.0), (1.27, 100.0), and (2.0, 200.0) respectively on the heat map A. ${\rm B}_1$, ${\rm B}_2$, ${\rm B}_3$, and ${\rm B}_4$ correspond to the time course for the parameter set (0.5,500.0), (1.3, 500.0), (0.96, 100.0), and (2.0, 200.0) respectively on the heat map B. And ${\rm C}_1$, ${\rm C}_2$, ${\rm C}_3$, and ${\rm C}_4$ for the parameter set (2.0,500.0), (4.0, 500.0), (2.0, 100.0), and (4.0, 100.0) respectively on the heat map C. First, and second term in the parameter set correspond to magnitude of stress (*I*), and $K_3$ respectively. Green, yellow, and red region indicate active, apoptotic, and cancer phase respectively on the heap map (**A**–**C**).

In this section, we study the dynamical behavior of $\hbox {p53}_{\rm A}$, and $\hbox {p53}_{\rm M}$ in two-parameter space driven by different stress (Fig. 6A). Each point in two-parameter space (*Magnitude* *of* *stress*, $K_3$) (Fig. 6A) is calculated concentrations of $p53_A$ in the dynamics: for oscillatory dynamics each point is the average of maxima and minima obtained in the time interval [145.82, 166.66] h, otherwise (no oscillation) concentration are measured at time 166.66 h. Figure 6 A (with constant stress) shows three distinct regimes/phases active (green region), apoptosis (yellow region) and cancer (red region). For large value of $K_3$, transition from active to cancer state is through apoptotic phase, while for low value of $K_3$, the range of apoptotic regime is so thin that slight increase in stress magnitude (*I*) might lead to direct cancer phase. Transition from active to apoptotic state is one directional. Figure 6$\hbox {A}_{1}$, $\hbox {A}_{2}$, $\hbox {A}_{3}$, and $\hbox {A}_{4}$ are the time course at different point on the heat map (Fig. 6A).

Similar behavior was observed in the patterns of two-parameter space in case of periodic stress (Fig. 6B). $\hbox {B}_{1}$, $\hbox {B}_{2}$, $\hbox {B}_{3}$, and $\hbox {B}_{4}$ show the corresponding time series for the parameter set (0.5,500.0), (1.3, 500.0), (0.96, 100.0), and (2.0, 200.0) respectively on the heat map. It is also observed that in the case of periodic stress, less magnitude of stress is required for different phase transition than constant stress.

In the case of exponentially decaying stress, we observed only two states active (green region), and cancer (red region) (Fig. 6C) unlike constant, and periodic stress. The significantly small yellow region as compared to active and cancer regions is observed in the phase diagram indicating either active state or cancer state (due to 166.66 h window). Further, the behavior also suggests that increasing the magnitude of stress signal, and decreasing $K_{3}$ parameter value enhances the chance of inducing cancer phase in the system dynamics. Hence, $K_3$ parameter is a crucial parameter for cancer dynamics where a low value of $K_3$ leads to more chances of having cancer^73^. Similar behavior was observed in case of c-Myc as oncogene for constant, oscillatory, and decaying stress Fig. S3A,B,C respectively.

The results discussed above indicate that apart from different stress, introduced in the system, there are various other factors which can drive the system to cancer state, for example, oncogene and its associated pathway/s. These factors are in fact, the key to sustain the system at an active state or bring back to active state from the pre-malignant state by regulating these parameters and their associated pathways. Moreover, the identification of these *critical point/s* and pre-malignant state is very important.

##### Cancer recovery phase: dynamics of pre-malignant state

**Figure 7:**
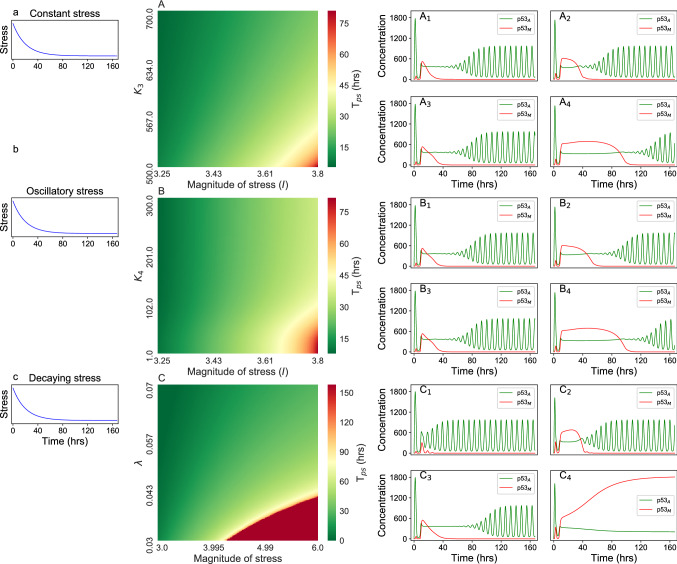
The left column show decaying stress. Second column, (**A**–**C**), show two parameter cancer recovery behavior for the parameter set (*magnitude* *of* *stress*, $K_{3}$), (*magnitude* *of* *stress*, $K_{4}$), and (*magnitude* *of* *stress*, $\lambda$) respectively driven with same decaying stress. ${\rm A}_1$, ${\rm A}_2$, ${\rm A}_3$, and ${\rm A}_4$ correspond to the time course for the parameter set (3.25,700.0), (3.8, 700.0), (3.25, 500.0), and (3.8, 500.0) respectively on the heat map A. ${\rm B}_1$, ${\rm B}_2$, ${\rm B}_3$, and ${\rm B}_4$ correspond to the time course for the parameter set (3.25,300.0), (3.8, 300.0), (3.25, 1.0), and (3.8, 1.0) respectively on the heat map B ($K_{3}=500.0$). ${\rm C}_1$, ${\rm C}_2$, ${\rm C}_3$, and ${\rm C}_4$ correspond to the time course for the parameter set (3.0,0.07), (6.0,0.07), (3.0,0.03), and (6.0,0.03) respectively on the heat map C ($K_{3}=1000.0$, $K_{4}=10.0$). On the heat map green color shoes lowest recovery time, while red shows highest recovery time or no recovery (in case of cancer).

In this section we focus on the properties of the *pre-malignant*, and *critical point/s*, and their importance in therapeutic intervention to prevent the cancer. As we have discussed in previous sections, we could able to find only one critical point ($T_c$) for constant, and periodic stress driven system (Figs. 2, 4 and 6). In these cases the pre-malignant state is just the beginning of cancer state, and it is hard to bring back to normal state. The scenario is quite different for exponentially decay stress. Here, we study the recovery time behavior for three different set of paramaters such as (*magnitude* *of* *stress*, and $K_{3}$), (*magnitude* *of* *stress*, and $K_{4}$), and (*magnitude* *of* *stress*, and $\lambda$) (Fig. 7). In this case, we observed two critical points $T_{c_1} > 0$ and $T_{c_2} > 0$ with $T_{c_2} > T_{c_1}$ separated by a time interval $T_{ps} = T_{c_2} - T_{c_1} \ge 0$ in the $\hbox {p53}_{\rm A}$ and $\hbox {p53}_{\rm M}$ dynamics. However, for $time < T_{c_1}$ and $time > T_{c_2}$, the system dynamics will be in active state, where $\hbox {p53}_{\rm A}$ dynamics showed sustain oscillatory behavior controlling $\hbox {p53}_{\rm M}$ dynamics to maintain at minimum concentration level ($\hbox {p53}_{\rm M}$ < $\hbox {p53}_{\rm A}$). This particular state is termed as pre-malignant state (discussed earlier), and is shown in Fig. 7. For certain values of the parameter set we observed that the system dynamics show a situation, $T_{ps}\rightarrow \infty$, $T_{c_1}\rightarrow T_{c_2}\rightarrow T_c$ and $\hbox {p53}_{\rm M}$ > $\hbox {p53}_{\rm A}$ exhibit stable attractor, then the dynamical system becomes *cancer* state. In this case, we did not observed *apoptotic state*.

We observe that by decreasing $K_3$ and increasing the magnitude of stress *I*, $T_{ps}$ is increased, but $T_{c_{1}}\rightarrow constant (same)$, which is *pre-malignant* state (Fig. 7*A*) in the parameter space of *I*, and $K_3$ (Fig. S3D for c-Myc as oncogene). In such situation, there is always a possibility of bringing back into active state. However, for significantly small $K_3 \le K_3^c$ and large $I \ge I^c$, where, $K_3^c$ and $I^c$ being critical values, we could able to observe the cancer state condition: $\displaystyle \lim _{(K_3\le K_3^c,I\ge I^c)}T_{ps}\rightarrow \infty$, $T_{c_1}\rightarrow T_{c_2}\rightarrow T_c$ and $p53_M > p53_A$ exhibiting stable attractor. Once the system reaches this phase, the dynamical process of the system becomes irreversible, and the system could not back to active state. Similar behavior and dynamical patterns can be found for set of (*I*, and $K_4$) in Fig. 7B and $B_1-B_4$, and for set of (*I*, $\lambda$) in Fig. 7C, $C_1-C_4$, where we could see the three states distinctly. Further, the coexistence of $\hbox {p53}_{\rm A}$ and $\hbox {p53}_{\rm M}$ may correspond to the point of intersection of normal and mutant p53 dynamics as in Fig. 6 ($\hbox {p53}_{\rm A}\, \sim \, \hbox {p53}_{\rm M}$) or the range between two such intersecting points as in Fig. 7 ($|\hbox {p53}_{\rm A}-\hbox {p53}_{\rm M}|\, \sim$ constant), such that, the normal oscillations in the dynamics of the $\hbox {p53}_{\rm A}$ become lost which could be due to loss of normal functioning. Once the system cross this range of coexistence the $\hbox {p53}_{\rm M}$ regain its normal oscillation (Fig. 7). The cancer and normal phases may correspond to when $\hbox {p53}_{\rm A}$ $<<$ $\hbox {p53}_{\rm M}$; and $\hbox {p53}_{\rm A}$ $>>$ $\hbox {p53}_{\rm M}$ respectively.

From the perspective of dynamical system analysis, identification of these three states obtained in any kind of cancer is quite important in view of prevention from that cancer. The reason could be due to the possibility of bringing back to normal condition from pre-malignant signature. Proper therapeutic intervention and drug administration needed to be done during the time $T_{ps}$ to prevent from cancer phase. It may not be able to cure cancer once the proper intervention and preventive measures are not taken up. Further, for the sake of cancer drug delivery, this pre-malignant state could be the proper stage of an investigation.

## Discussion and summary

In therapeutic intervention, cancer can be treated broadly in two ways by exploring dynamical behavior along with hidden patterns of cancer and associated cellular states, and second to explore proper cellular state and time for therapeutic intervention or drug discovery. In the present work, we have studied a model that incorporates the dynamics of both active and mutant p53 that are driven by different forms of time-dependent stress. We have considered the impact of ARF and oncogenes through different feedback mechanisms. This simple model has four distinct final states that can be characterised by the asymptotic dynamics: these have experimental validation^29,75^ and variously correspond to active, apoptotic, pre-malignant and cancer states.

A dynamical systems approach can offer fresh insights to understanding cancer progression, and therefore suggest new protocols. Sustained oscillations in ${\rm p}53_A$ and ${\rm p}53_M$ dynamics can be seen as repeated pulses that occur in the system when DNA damage is repaired. Such oscillations persist until the DNA repair is completed^33^. Stress that triggers the system to the active state is a reversible process, the dynamics reverting to normal when the stress is removed. For high stress or when there are $p53_M$ activators such as oncogene or ARF, the amplitude of ${\rm p}53_A$ oscillation with being large enough to arrest the cell cycle. In this situation, the amplitude of $p53_M$ reaches a critical level, although lower than the amplitude of ${\rm p}53_A$^76^. Oscillatory dynamics vanishes as stress crosses a certain threshold^66^ for both ${\rm p}53_A$ and ${\rm p}53_M$; this is a state of amplitude death leading to a stable fixed-point attractor. This corresponds to apoptosis since the system cannot revert to oscillatory dynamics: this is an irreversible transition^25^.

For large stress, the production of mutant ${\rm p}53_M$ becomes rapid and uncontrolled. The concentration level of ${\rm p}53_M$ exceeds a critical apoptotic threshold, and this can be seen as stress-induced premature senescence. This suppresses apoptosis and triggers cancer progression^77,78^. For constant or a periodic stress signal, we were able to find a condition where ${\rm p}53_A$ and ${\rm p}53_M$ coincide. We term this a critical point of the dynamical system, and this can be considered as leading to a new cancer state: mutant ${\rm p}53_M$ is uncontrollable ($p53_M\rangle p53_A$). Furthermore, there is no possibility of DNA repair, and the process is irreversible. However, there is a small range of stress where the concentration of mutant p53 increases slowly, compared to the monotonic increase in the cancer regime. This we term as pre-malignant. For constant or periodic stress, there is a single critical point and hence the system, having transitioned to the cancer state *cannot* revert to the normal state.

For exponentially decaying stress, only three states can be observed: active, pre-malignant or cancer. There are two critical points, in this case, indicating the possibility of reversing from the pre-malignant to the active state. The width of the transition region depends on the stress-inducing parameters with respect to oncogene, ARF, and other mechanisms. Identification of this range of the pre-malignant state, along with critical points, is important for therapeutic intervention.

Our study provides a qualitative picture of the dynamical properties of states observed in various experiments on cellular dynamics.The present results indicate the possibility of measuring how much stress suffices to lead to cancer. It will be important to explore the role of noise in driving the dynamics to see how robust these results are to extrinsic or intrinsic stochasticity.

## Supplementary Information

Supplementary material 1.
