# Supplementary material for: Transition and identification of pathological states in p53 dynamics for therapeutic intervention
Source: Sci Rep. 2021 Jan 27;11:2349. doi: 10.1038/s41598-021-82054-1 (PMC7840995; doi:10.1038/s41598-021-82054-1)
Supplement: Supplementary file 1 — Supplementary material 1. [file 41598_2021_82054_MOESM1_ESM.pdf]

Supplementary Information

Transition and Identification of Pathological States in p53 Dynamics for Therapeutic Intervention

Amit Jangid[1,2], Md Zubbair Malik[1], Ram Ramaswamy[2] and R.K. Brojen Singh[1]
[1] *School of Computational and Integrative Sciences, Jawaharlal Nehru University, New Delhi 110067, India.*
[2] *Department of Chemistry, Indian Institute of Technology Delhi, New Delhi 110016, India.*

Minimal regulatory network

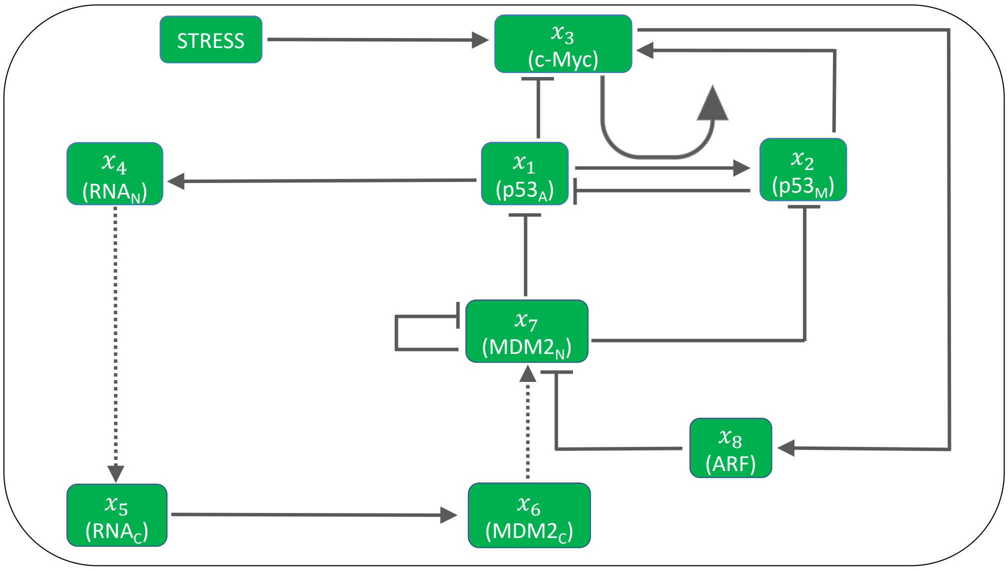

FIG. S1: Interaction network for $p53_A$-$p53_M$-MDM2-ARF-Stress. Modified network from [1] where $p53_A$ inhibits activation of c-Myc [2] [3]. c-Myc pro-oncogene induces the expression of $p53_M$ from $p53_A$ due to de-ragulation in c-Myc [4]. Dashed arrow shows movement from nucleus to cytoplasm or vice versa, while solid arrow, and bars corresponds to activation, and inhibition on respective node.

Mathematical framework of the model system

$$
\begin{aligned}
\frac{dx_1}{dt} &= k_p - \left(k_1\,x_7 + d_p + \gamma_{_{x_1}}\,x_2 + \delta_{_{x_1}}\,\frac{x_3^{n_1}}{K_1^{n_1} + x_3^{n_1}}\right)x_1 \\
\frac{dx_2}{dt} &= \alpha_{x_2} + \delta_{x_1}\,\frac{x_3^{n_1}}{K_1^{n_1} + x_3^{n_1}}\,x_1 - \gamma_{x_2}\,x_7\,x_2 - \delta_{x_2}\,x_2 \\
\frac{dx_3}{dt} &= \alpha_{x_3} + \beta_{x_3}\,\frac{S^{n_2}}{K_2^{n_2} + S^{n_2}} + \delta_{x_3}\,\frac{{x_2}^{n_3}}{K_3^{n_3} + {x_2}^{n_3}} - \gamma_{x_3}\,x_3 - \omega_{_{x_3}}\,x_3\,x_1 \\
\frac{dx_4}{dt} &= k_m + k_2\,\frac{x_1^{1.8}}{k_D^{1.8} + x_1^{1.8}} - k_0\,x_4 \\
\frac{dx_5}{dt} &= k_0\,x_4 - d_{rc}\,x_5 \\
\frac{dx_6}{dt} &= k_T\,x_5 - k_i\,x_6 \\
\frac{dx_7}{dt} &= k_i\,x_6 - d_{mn}\,x_6^2 - k_3\,x_7\,x_8 \\
\frac{dx_8}{dt} &= k_a + \delta\,\frac{x_3^{n_4}}{K_4^{n_4} + x_3^{n_4}}\,x_8 - d_a\,x_8 - k_3\,x_7\,x_8 \qquad (1)
\end{aligned}
$$

Here, x_3 is c-Myc oncogene, and $\omega_{_{x_3}}$ is the parameter which represents activated p53 dependent decay rate in c-Myc. $\omega_{_{x_3}}$ $= 9.963 \times 10^{-8}$, and rest of all the parameter are the same as in table 1 in main text. See the main text for the detail of the equations. Term, $\omega_{_{x_3}}\,x_3\,x_1$, in coupled differential equation shows the inhibition in c-Myc by $\mathrm{p53_A}$.

Results

Oncogenic regulation of normal and cancer dynamics

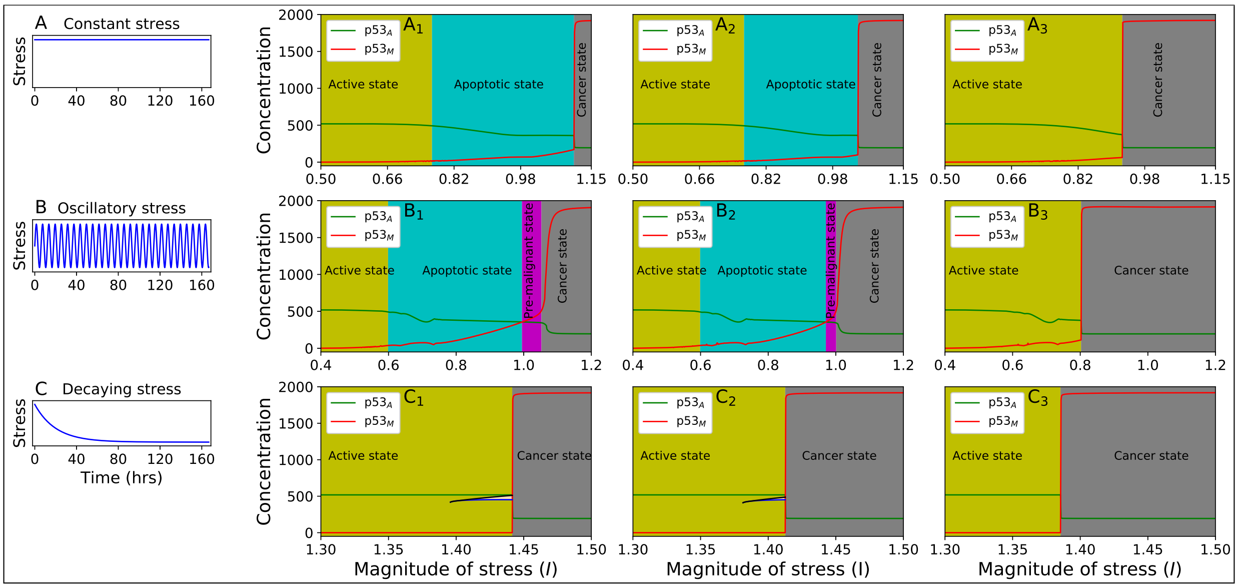

FIG. S2: The left column show three different form of stress discussed about. A_1, A_2, and A_3 display the steady state behaviour against magnitude of stress for different K_3 values 1000.0, 750.0, and 400.0 respectively driven with constant stress. B_1, B_2, and B_3 display the steady state behaviour against magnitude of stress for different K_3 values 2000.0, 1400.0, and 800.0 respectively driven with oscillatory stress. C_1, C_2, and C_3 display the steady state behaviour against amplitude for different K_3 values 700.0, 650.0, and 600.0 respectively driven with decaying stress. Yellow region, cyan region, and grey region correspond to active, apoptotic, premalignant, and cancer state respectively. In panel C_1, and C_2 (wheat region) black line (upper line), and blue line (lower line) show maximum of $p53_M$, and maximum of $p53_A$ in T_{ps} (see the text) time region, which corresponds to the initial cancer condition. In constant stress case we did not observe pre-malignant regime.

Phase transition, key to therapeutic intervention and cancer recovery phase

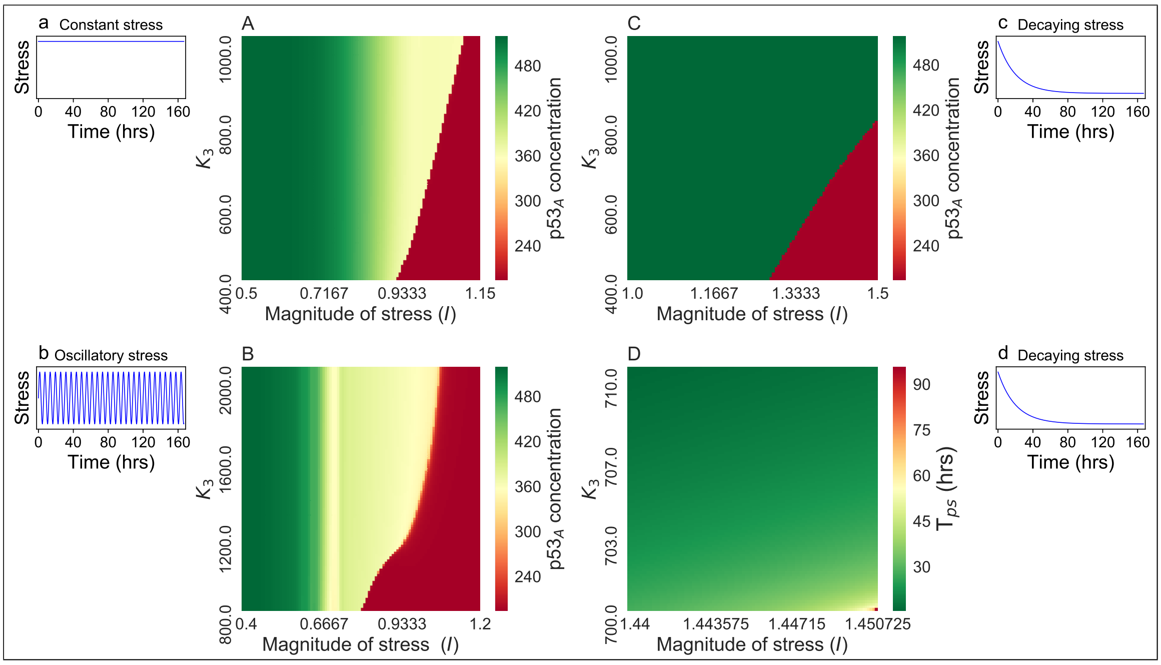

FIG. S3: A, B, and C show the two parameter ($Magnitude\ of\ stress$ (I), K_3) steady state behavior of the system driven by different stress a, b, and c respectively. D shows two parameter cancer recovery behaviour of the system ($magnitude\ of\ stress$, and K_3) driven with decaying stress. On the heap map (panel A, B, and C) green, yellow, and red region indicate active, apoptotic, and cancer phase respectively.

[1] G. B. Leenders and J. A. Tuszynski. Stochastic and deterministic models of cellular p53 regulation. *Frontiers in oncology*, **3**, (2013).

[2] J. S. L. Ho, W. Ma, D. Y. L. Mao, and S. Benchimol. p53-dependent transcriptional repression of c-myc is required for g1 cell cycle arrest. *Molecular and Cellular Biology*, **25**, 7423–7431, (2005).

[3] M. Sachdeva et al. Mo. p53 represses c-myc through induction of the tumor suppressor mir-145. *Proceedings of the National Academy of Sciences*, **106**, 3207–3212, (2009).

[4] B. Roy, J. Beamon, E. Balint, and D. Reisman. Transactivation of the human p53 tumor suppressor gene by c-myc/max contributes to elevated mutant p53 expression in some tumors. *Molecular and Cellular Biology*, **14**, 7805–7815, (1994).
